# Extrahepatic portosystemic shunt concealed in congenital heart disease and neurodevelopmental disorder: a case report

**DOI:** 10.1093/ehjcr/ytaf135

**Published:** 2025-03-25

**Authors:** Toshinobu Ifuku, Hazumu Nagata, Yusaku Nagatomo, Ichiro Sakamoto, Keigo Nakatani

**Affiliations:** Department of Pediatrics, Miyazaki Prefectural Miyazaki Hospital, 5-30, Kita Takamatsu-cho, Miyazaki, Miyazaki 880-8510, Japan; Department of Pediatrics, Graduate School of Medical Sciences, Kyushu University, 3-1-1, Maidashi, Higashi-ku, Fukuoka, Fukuoka 812-8582, Japan; Department of Pediatrics, Graduate School of Medical Sciences, Kyushu University, 3-1-1, Maidashi, Higashi-ku, Fukuoka, Fukuoka 812-8582, Japan; Department of Cardiovascular Medicine, Graduate School of Medical Sciences, Kyushu University, 3-1-1, Maidashi, Higashi-ku, Fukuoka, Fukuoka 812-8582, Japan; Department of Pediatrics, Miyazaki Prefectural Miyazaki Hospital, 5-30, Kita Takamatsu-cho, Miyazaki, Miyazaki 880-8510, Japan

**Keywords:** Case report, Abernethy malformation, Congenital heart disease, Autism spectrum disorder, Intellectual disability

## Abstract

**Background:**

Neurodevelopmental disorders (NDDs) are often associated with congenital heart diseases (CHDs). Congenital portosystemic shunt (CPSS) is a rare abnormality of the portal system in which toxic substances that are not adequately metabolized by the liver circulate throughout the body and can cause non-specific neuropsychiatric symptoms. We describe a case of CHD and NDD in which neuropsychiatric symptoms due to extrahepatic CPSS became evident in adulthood.

**Case summary:**

A 24-year-old man underwent a thorough examination for liver dysfunction. He had a history of repaired tetralogy of Fallot and autism spectrum disorder. He was also diagnosed with depression at 21 years of age. Abdominal contrast-enhanced computed tomography revealed an abnormal vessel descending from the main trunk of the portal vein and entering the left common iliac vein, which was diagnosed as a CPSS. Hyperammonaemia, focal nodular hyperplasia of the liver, and high signal intensity in the bilateral globus pallidus on T1-weighted brain magnetic resonance imaging were also observed. Transcatheter occlusion of the CPSS with a multilayer device (Vascular Plug II; AGA Medical Corporation, Plymouth, MN, USA) significantly improved neuropsychiatric symptoms, abnormal blood data, and head and liver lesions.

**Discussion:**

Some of the neuropsychiatric symptoms in this patient were thought to have been caused by portosystemic encephalopathy (PSE) associated with CPSS. The symptoms of PSE and NDD are sometimes similar and difficult to differentiate. Although complications of CHD and NDD are common, screening for secondary treatable neuropsychiatric disorders, such as PSE, should be considered.

**Learning points**

- Congenital heart disease (CHD) is often complicated by neurodevelopmental disorders and requires support and care for the neuropsychiatric symptoms.
- Congenital portosystemic shunt (CPSS) is a rare abnormality of the portal venous system that sometimes leads to non-specific neuropsychiatric symptoms, called portal venous somatic encephalopathy (PSE). CPSS is often associated with CHD.
- Differentiating and ruling out secondary and treatable causes such as PSE for neuropsychiatric symptoms in patients with CHD is important.

## Introduction

Neurodevelopmental disorders (NDD) are often comorbid with congenital heart disease (CHD), with varying symptoms and severity. Neurodevelopmental disorders are caused by the complex interplay between congenital and acquired factors. Patients with CHD can develop psychiatric disorders later in adolescence, requiring physical and mental support and care.^1,2^

Congenital portosystemic shunt (CPSS) is a rare anomaly that occurs in 1 in 30 000–50 000 births. In CPSS, abnormal intrahepatic or extrahepatic shunt vessels connect the portal venous system to the systemic venous system, bypassing the hepatic circulation. Elevated levels of toxic substances, such as ammonia, in the systemic veins via CPSS may cause non-specific neuropsychiatric symptoms, called portosystemic encephalopathy (PSE), and brain, lung, and liver lesions. Congenital portosystemic shunt presents with diverse clinical manifestations, including asymptomatic cases, and is diagnosed at various ages.^3^ Congenital heart diseases are occasionally associated with CPSS, although these relationships remain unclear.^4^ In this paper, we describe a case of CHD and NDD in which neuropsychiatric symptoms due to extrahepatic CPSS became evident during adulthood.

## Summary figure

**Figure:**
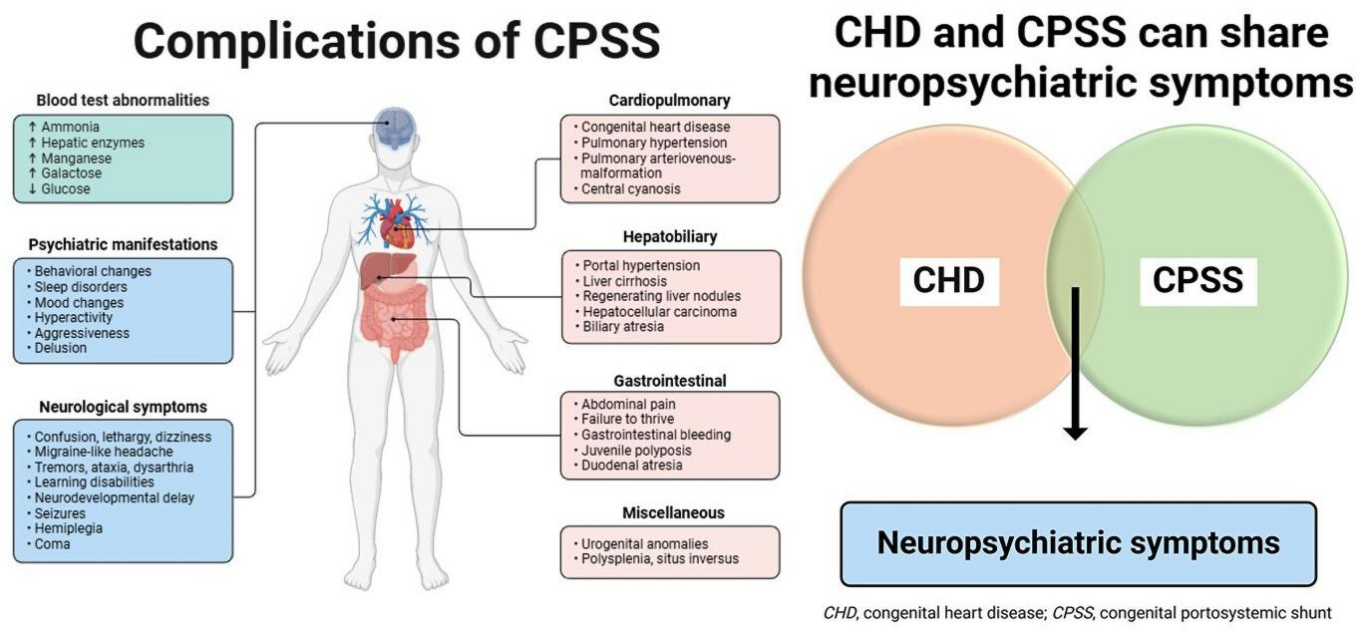


## Case presentation

A 24-year-old man, with persistently elevated serum gamma-glutamyl transpeptidase (γ-GTP) for the past 2 years, underwent a thorough examination. The patient was diagnosed with tetralogy of Fallot during the neonatal period. At 1 year of age, he underwent intracardiac repair with a transannular patch. Bioprosthetic pulmonary artery valve replacement was performed at ages 17 and 21 years for residual pulmonary artery stenosis and regurgitation. Aspirin was continued as antiplatelet therapy.

The patient was also noted to have developmental delays beginning in infancy, and was diagnosed with autism spectrum disorder during his school years. Around the age of 21 years, his mood swings, insomnia, fatigue, and slow reactions became more noticeable. A psychiatrist diagnosed the patient with depression and administered antidepressants (10–20 mg of escitalopram) and sleep-inducing drugs (5 mg of lemborexant), which failed to improve his symptoms. He was only able to visit a work facility 1–3 days a week. Chromosomal analysis revealed a normal male karyotype. No family history of CHD or genetic abnormalities was noted.

On examination, his vital signs were as follows: Glasgow Coma Scale score, 15 (E4V5M6); body temperature, 36.4°C; blood pressure, 106/50 mmHg; pulse rate, 65 beats per minute; respiratory rate, 12 breaths per minute; and oxygen saturation, 98%. Physical examination revealed systolic ejection murmur grade 2/6 at the mid-left sternal border. No obvious abdominal masses or hepatomegaly were observed. The echocardiogram showed a left ventricular ejection fraction of 62.4%, a right ventricular fractional area change of 53.1%, and mild pulmonary artery stenosis and regurgitation were present. There were no findings suggesting pulmonary hypertension. Blood tests confirmed elevated levels of ammonia, bile acids, and γ-GTP (*Table 1*).

**Table 1:** Initial laboratory data

Peripheral blood tests		(Normal range)	Biochemistry tests		(Normal range)	LDH	212	124–222 IU/L
WBC	4.03	3.30–8.60 × 10^3^/μL	TP	6.0	6.6–8.1 g/dL	γ-GTP	70	13–64 IU/L
Neu	51.6	40.0–70.0%	Alb	3.4	4.1–5.1 g/dL	Glucose	111	73–109 mg/dL
Ly	32.7	18.0–53.0%	BUN	9.0	8.0–20.0 mg/dL	Bile acid	201.3	1.3–9.0 μmol/L
RBC	5.14	4.35–5.55 × 10^6^/L	Cr	0.54	0.65–1.07 mg/dL	BNP	42.7	≤18.4 pg/mL
Hb	15.4	13.7–16.8 g/dL	UA	4.8	3.7–7.8 mg/dL	NH3	264	12–66 μg/dL
Ht	47.8	40.7–50.1%	Na	142	138–145 mEq/L	AFP	1.4	0.0–10.0 ng/mL
MCV	93.1	83.6–98.2 fL	K	3.8	3.6–4.8 mEq/L	CA19–9	7.1	≤37.0 U/mL
Platelet	196	158–348 × 103/μL	Cl	107	101–108 mEq/L	Mn	2.4	0.8–2.5 μg/dL

Coagulation study		(Normal range)	Ca	8.7	8.8–10.1 mg/dL			
PT	13.6	10.0–13.5 s	T-Bil	1.40	0.4–1.5 mg/dL			
APTT	37.8	26.0–41.0 s	AST	37	13–30 IU/L			
PT-INR	1.20	0.90–1.10	ALT	44	10–42 IU/L			
D-dimer	1.10	0.0–1.0 μg/mL	CPK	146	59–248 IU/L			

WBC, white blood cell; Neu, neutrophils; Ly, lymphocytes; RBC, red blood cell; Hb, haemoglobin; Ht, haematocrit; MCV, mean corpuscular volume; PT, prothrombin time; APTT, activated partial thromboplastin time; PT-INR, prothrombin time-international normalized ratio; TP, total protein; Alb, albumin; BUN, blood urea nitrogen; Cr, creatinine; UA, uric acid; Na, sodium; K, potassium; Cl, chlorine; Ca, calcium; T-Bil, total-bilirubin; AST, aspartate aminotransferase; ALT, alanine aminotransferase; CPK ,creatinine phosphokinase; LDH, lactase dehydrogenase; γ-GTP, γ-glutamyl transpeptidase; BNP, brain natriuretic peptide; AFP, α-fetoprotein; CA19-9, carbohydrate antigen19-9; Mn, manganese.

**Figure 1:**
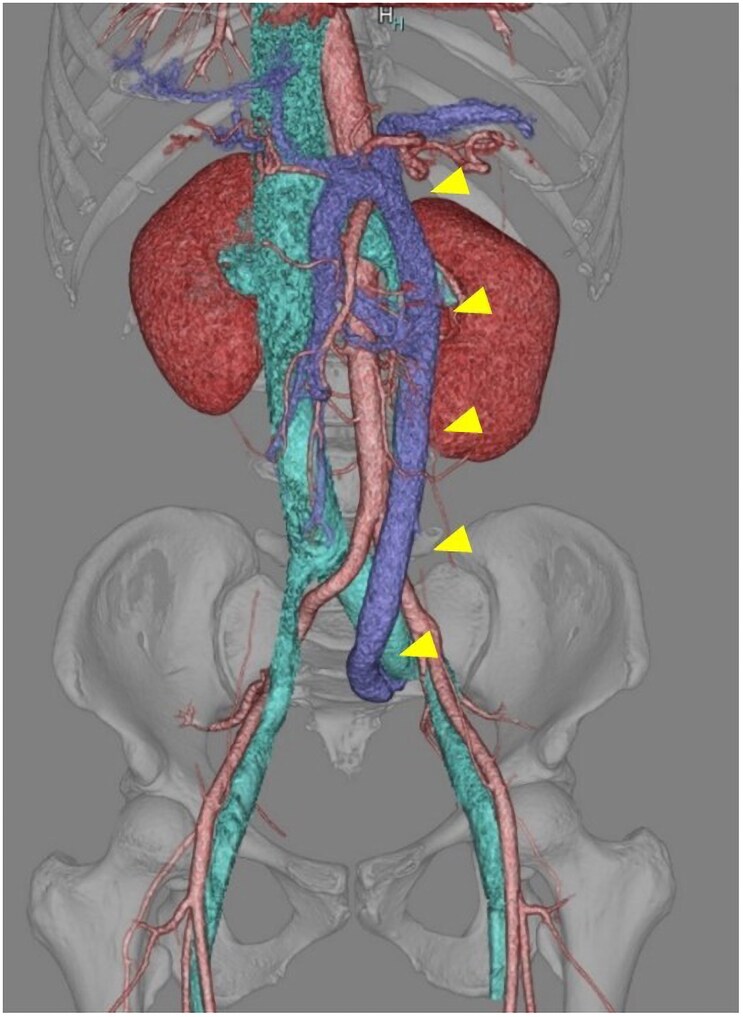
3D reconstructed abdominal contrast-enhanced computed tomography. The congenital portosystemic shunt (CPSS; arrowheads) descends from the main portal vein trunk and flows into the left common iliac vein.

**Figure 2:**
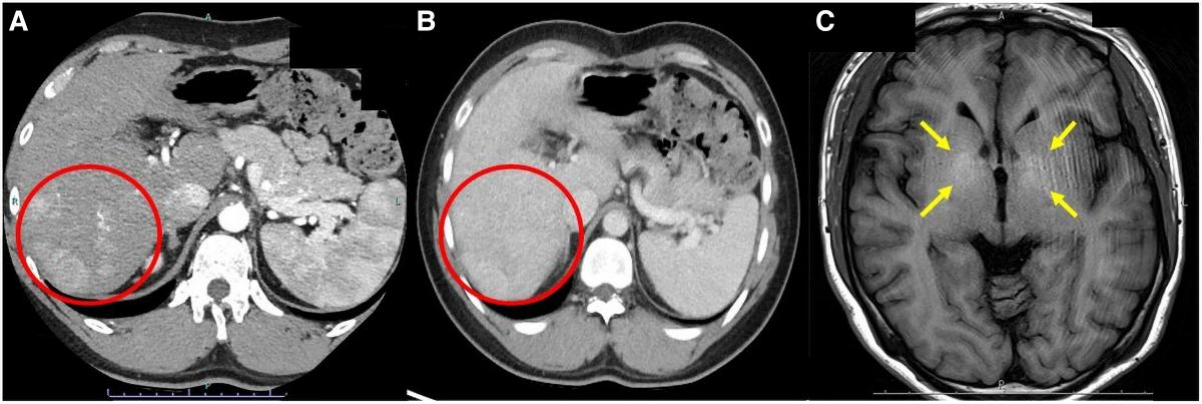
(*A* and *B*) Abdominal contrast-enhanced computed tomography shows multiple focal nodular hyperplasia within the liver (circle). (*A*) Focal nodular hyperplasia shows mostly homogeneous strong staining in the arterial phase. (*B*) In the portal phase, the contrast staining of FNH has disappeared. (*C*) Brain magnetic resonance imaging shows high signals in the bilateral globus pallidus on T1-weighted images (arrows).

Abdominal ultrasonography revealed multiple liver masses with a combination of hypoechoic and isoechoic structures. Abdominal contrast-enhanced computed tomography (CT) revealed an abnormal vessel descending from the main portal vein trunk and entering the left common iliac vein (*Figure 1*). Multiple liver masses showed mostly homogeneous strong staining in the arterial phase, which disappeared in the portal venous phase, a finding consistent with a diagnosis of focal nodular hyperplasia (FNH) (*Figure 2A* and *B*). Brain magnetic resonance imaging (MRI) confirmed high signal intensity in the bilateral globus pallidus on T1-weighted images (*Figure 2C*).

**Figure 3:**
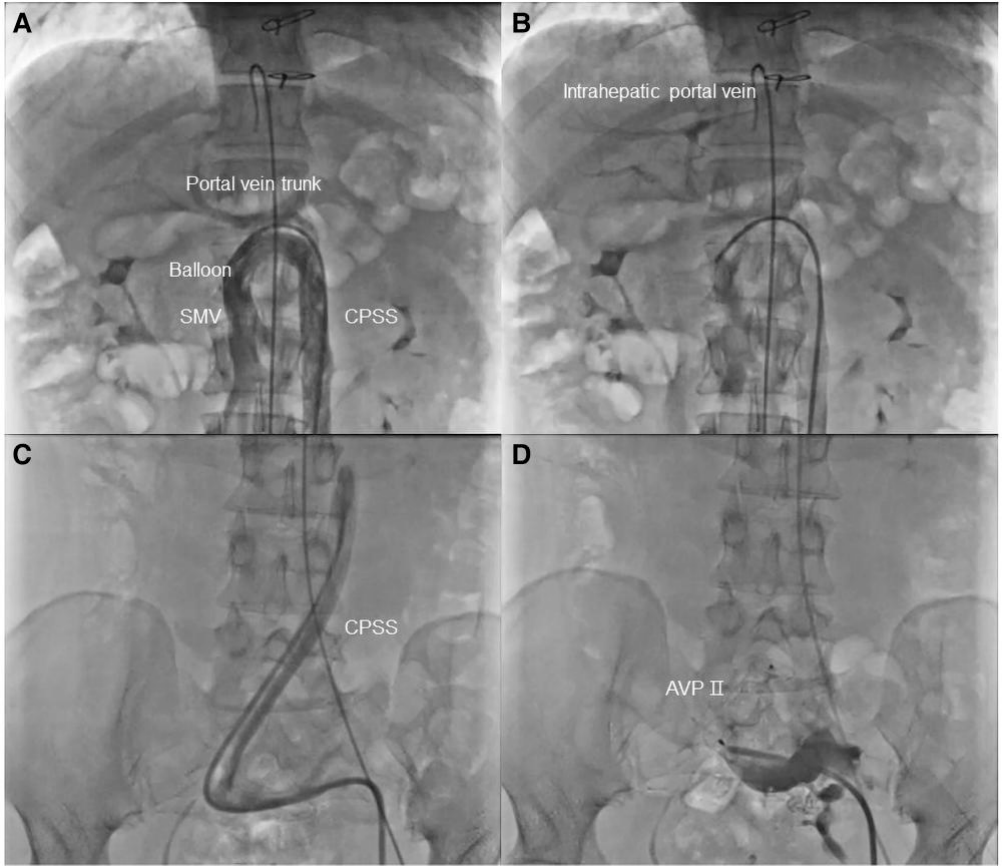
(*A*) Selective occlusion balloon angiography in the superior mesenteric vein. (*B*) The intrahepatic portal vein branches are relatively well-developed. (*C* and *D*) Transcatheter occlusion of the congenital portosystemic shunt using a 20-mm AMPLATZER Vascular Plug II (AVP II; AGA Medical Corporation, Plymouth, MN, USA). CPSS, congenital portosystemic shunt.

Given that CPSS was the suspected cause of the brain and liver lesions and blood data abnormalities, we decided to evaluate the haemodynamics and determine the indications for treatment via cardiac catheterization. Left femoral vein access was achieved as the most linear route through the CPSS and portal venous system. Portal vein pressure was measured as 9 mmHg before and 15 mmHg after balloon occlusion. Selective occlusion balloon angiography showed that the intrahepatic portal vein branches were relatively well-developed (*Figure 3A* and *B*). The mean central venous pressure was 7 mmHg and the main pulmonary artery pressure was 14 mmHg. Pulmonary arteriography revealed no pulmonary arteriovenous malformations. Based on these results, transcatheter occlusion of the CPSS was achieved by using a 20-mm Amplatzer Vascular Plug II (AGA Medical Corporation, Plymouth, MN, USA). The plug size was based on the diameter of the CPSS in the target segment obtained by using CT and angiography. While evaluating the angiogram, the device was implanted to avoid the area where the intestinal vein flowed into the shunt vessel, and complete occlusion of the shunt was confirmed (*Figure 3C* and *D*).

Blood ammonia levels normalized promptly after treatment, and other abnormal blood data improved within a few weeks. Of note was the dramatic improvement in neuropsychiatric symptoms, with the patient becoming noticeably more active and less tired and fatigued. The responses to conversations and actions were also quick. The patient was able to work almost daily with a reduced dose of antidepressants (5 mg of escitalopram). Furthermore, he has been able to sleep without sleep medication. Six months after treatment, abdominal CT and head MRI images showed a reduction in FNH and the disappearance of abnormal signals on T1-weighted images, respectively.

## Discussion

In this paper, we reported a case of tetralogy of Fallot with CPSS which was treated in adulthood. He was diagnosed with NDD and depression. Some symptoms improved after CPSS occlusion. In patients with CHD, many factors are considered to have a role in the risk of NDD, including genetics, CHD structure, haemodynamics, and the prenatal/postnatal environment. Compared with the general population, patients with CHD have a higher risk of anxiety and depression in childhood, as well as a higher prevalence of psychiatric concerns in adulthood.^1,2^

The exact aetiology of CPSS remains unknown and a causative gene has not yet been identified. Remnant or excessive retraction of the left and right vitreous veins during foetal life may cause CPSS.^3,5^ Since a CPSS bypasses the liver and enters the systemic circulation, ammonia, bile acids, and manganese fail to undergo adequate first-pass metabolism in the liver, thereby elevating their concentration in the peripheral blood. We speculate that the hyperplastic response to abnormal vascular and blood flow in the liver triggers FNH and that the deposition of manganese in the brain parenchyma causes a high signal in T1-weighted MRI.^3,6^

The diagnostic triggers for CPSS vary and include abnormal liver function tests, hypergalactosaemia, biliary stasis, failure to thrive, incidental findings during the workup of a liver mass, pulmonary hypertension, or other congenital defects. In recent years, prenatally diagnosed cases of CPSS have been reported.^7,8^ Congenital heart diseases are associated with 17–23% of CPSS cases (*Table 2*).^3,4,9^ Some researchers believe that foetal haemodynamic abnormalities due to CPSS are partially responsible for the development of CHD.^3^ While the pathophysiology is not fully elucidated, CPSS can cause heart failure, pulmonary hypertension, and cyanosis itself. When CPSS is complicated by complex or severe CHD, its management often becomes difficult.^4^ Although the patient had undergone three cardiac surgeries, detailed imaging examinations of the brain and abdomen were not performed. These findings suggest that when treating patients with CHD, it is necessary to pay attention to symptoms and abnormal test data other than cardiovascular system, and to differentiate the complications of CPSS.

**Table 2:** The association between CPSS and cardiovascular disease^3,4^

Congenital heart disease	Symptoms
Atrial or ventricular septal defect	Heart failure
Patent foramen ovale	Central cyanosis
Patent ductus arteriosus	Portopulmonaryhypertension
Congenital stenosis of aortic or pulmonary valves	Hepatopulmonary syndrome
Tetralogy of Fallot	Neonatal pulmonary hypertension
Single ventricle	Isolated neonatal respiratory distress
Tricuspid or mitral atresia	
Transposition of the great arteries	
Coarctation of the aorta	
Miscellaneous	
Pulmonary arteriovenous malformation	
Heterotaxy syndrome, left isomerism	
Isolated situs inversus	

In 17–30% of children with CPSS, systemic ammonia and other toxic substances have been reported to cause neuropsychiatric symptoms, known as PSE, which is more likely to occur with age and increased shunt volume.^3,10^ Non-specific symptoms such as fatigue, intellectual disability, and behavioural disturbances may be present.^6^ We believe that some NDD and depressive symptoms were attributable to PSE in the present patient. Increased shunt volume with age may exacerbate neuropsychiatric symptoms and liver dysfunction. The intrahepatic portal vein was fortunately well-developed on angiography, and no portal or pulmonary hypertension existed, thereby allowing primary occlusion of the CPSS.

The neuropsychiatric symptoms of PSE and NDD are often non-specific and similar, making their differentiation difficult. Although complications of CHD and NDD are common, this study highlights the importance of screening for secondary, treatable neuropsychiatric disorders such as PSE. We hope that further findings will be accumulated, not only on the association between CPSS and CHD, but also from the perspective of neurodevelopmental science and psychiatry.

## Data Availability

Data sharing is not applicable to this article as no datasets were generated or analysed in the current study. The data are available from the corresponding author upon request.

## Lead author biography

Dr Toshinobu Ifuku graduated from Kumamoto University, Faculty of Medicine in 2009, trained at Fukuoka Children’s Hospital and now works as a Paediatric Cardiologist at Miyazaki Prefectural Miyazaki Hospital in his home town. He provides medical care for patients of all ages, from newborns to adults with congenital heart disease.
